# A glycosyl transferase family 43 protein involved in xylan biosynthesis is associated with straw digestibility in *Brachypodium distachyon*

**DOI:** 10.1111/nph.15089

**Published:** 2018-03-25

**Authors:** Caragh Whitehead, Francisco J. Ostos Garrido, Matthieu Reymond, Rachael Simister, Assaf Distelfeld, Sergio G. Atienza, Fernando Piston, Leonardo D. Gomez, Simon J. McQueen‐Mason

**Affiliations:** ^1^ Centre for Novel Agricultural Products Department of Biology University of York PO Box 373 Wentworth Way York YO10 5DD UK; ^2^ Departamento de Mejora Genética Vegetal Instituto de Agricultura Sostenible – Consejo Superior de Investigaciones Científicas Córdoba Spain; ^3^ Institut Jean‐Pierre Bourgin UMR 1318 INRA‐AgroParisTech INRA Centre de Versailles‐Grignon Route de Saint‐Cyr 78026 Versailles France; ^4^ Deparment of Molecular Biology and Ecology of Plants Tel Aviv University Tel Aviv Israel

**Keywords:** biomass, Brachypodium, gene silencing, GT43, saccharification, xylan

## Abstract

- The recalcitrance of secondary plant cell walls to digestion constrains biomass use for the production of sustainable bioproducts and for animal feed.
- We screened a population of Brachypodium recombinant inbred lines (RILs) for cell wall digestibility using commercial cellulases and detected a quantitative trait locus (QTL) associated with this trait.
- Examination of the chromosomal region associated with this QTL revealed a candidate gene that encodes a putative glycosyl transferase family (GT) 43 protein, orthologue of IRX14 in Arabidopsis, and hence predicted to be involved in the biosynthesis of xylan. Arabinoxylans form the major matrix polysaccharides in cell walls of grasses, such as Brachypodium. The parental lines of the RIL population carry alternative nonsynonymous polymorphisms in the *Bd* GT43A gene, which were inherited in the RIL progeny in a manner compatible with a causative role in the variation in straw digestibility. In order to validate the implied role of our candidate gene in affecting straw digestibility, we used RNA interference to lower the expression levels of the *Bd* GT43A gene in Brachypodium.
- The biomass of the silenced lines showed higher digestibility supporting a causative role of the *Bd* GT43A gene, suggesting that it might form a good target for improving straw digestibility in crops.

## Introduction

Global commitments to reducing carbon emissions, combined with concerns over food security are increasing the imperative for producing sustainable low‐carbon biofuels based on nonfood biomass, such as cereal straw or energy grasses (Marriott *et al*., 2016). Lignocellulosic biomass is largely composed of polysaccharides, which can be depolymerized using biochemical methods to provide sugars for conversion to biofuels via fermentation. However, the recalcitrant nature of lignocellulose to digestion stands as a significant barrier to the cost‐effective production of biofuels (Gomez *et al*., 2008; Hoekman, 2009; Naik *et al*., 2010; Marriott *et al*., 2016). Biomass recalcitrance also limits the value of crop residues as animal feed. Reducing biomass recalcitrance without having a negative impact on yield is therefore an important target for improving the value of crop residues for feed and biorefinery applications.

The main components of the secondary cell walls in grasses are cellulose (35–45%), hemicellulose (40–50%) and lignin (*c*. 20%) (Marriott *et al*., 2016). Complex arabinoxylans (AX) found in the hemicellulosic fraction of the cell wall form the major component of matrix polysaccharides in grasses and comprised a β, 1‐4 linked xylopyranose backbone that is decorated with side chains of arabinose, xylose, galactose and glucuronic acid, as well as acetyl groups (York & O'Neill, 2008; Scheller & Ulvskov, 2010; Busse‐Wicher *et al*., 2016).

The synthesis of the xylan backbone was first reported in Arabidopsis and it is thought that the xylan backbone of AX in grasses is synthesized in a similar fashion by a complex of glycosyltransferases (GTs) (Brown *et al*., 2007). The Arabidopsis xylan synthase complex contains three different GT proteins belonging to the GT8, GT43 and GT47 families (Rennie & Scheller, 2014). It is thought that the GT43 enzymes, IRX9 and 14, together with the GT47 protein, IRX10 are involved in the elongation of the xylan backbone (Brown *et al*., 2007). It has been shown that these three proteins work together in a complex in wheat (Zeng *et al*., 2010). However, it proved difficult to identify the specific function of each protein in xylan elongation until 2014 when Urbanowicz *et al*., 2014; confirmed biochemically that IRX10 has β‐1,4‐xylan synthase activity. It remains unclear if the GT43 genes have a catalytic role in terms of forming the xylan backbone, but their presence is required for the proper functioning of the complex (Ren *et al*., 2014). The Arabidopsis genome encodes four GT43 genes, namely, IRX9 and IRX14 and their homologs IRX9L and IRX14L, which are functionally nonredundant in the formation of the xylan backbone (Wu *et al*., 2010). However, in rice, 10 GT43 genes have been identified, *Os*GT43A–J (Lee *et al*., 2014). In 2013, Chiniquy *et al*. reported that three genes, *Os*GT43C, OsGT43F and *Os*GT43J, found in rice are putative orthologues to the Arabidopsis GT43 family genes IRX9, IRX9L and IRX14, respectively. Lee *et al*. (2014) studied four rice GT43 genes and discovered that *Os*GT43A and *Os*GT43E are orthologues for IRX9 and confirmed that *Os*GT43J is an orthologue of IRX14. They also suggested that *Os*GT43H was not an orthologue of either IRX9 or IRX14.

Grass AX contains more complex side chain decorations than that of xylans in dicot plants. Grass AX is dominated by α‐1,2‐ and α‐1,3‐linked arabinosyl side chains, whereas dicots predominantly contain glycuronosyl and 4‐*O*‐methylglucuronosyl residues as side chains (Mitchell *et al*., 2007; Chiniquy *et al*., 2012). It was recently shown that these arabinosyl side chains are added to the backbone by the action of GT61 proteins. The XAT1 and XAT2 genes belong to clade A of the large GT61 family of genes in Arabidopsis and are reported to have α‐1,3‐arabinosyltransferase activity. Some of the arabinosyl side chains are further decorated with a xylose unit added at position O‐2 by a β‐1,2‐xylosyltranserase, which is also thought to belong to the GT61 family (Anders *et al*., 2012). In rice, the GT61 gene XAX1 appears to be responsible for this addition, with loss of function resulting in lower concentrations of xylose and ferulic acid in the AX, accompanied by increased biomass digestibility (Chiniquy *et al*., 2012). Some arabinosyl residues are further decorated with ferulic acid (FA, *c*. 4%) or *p*‐coumaric acid (*p*CA *c*. 3%) (Hatfield *et al*., 1999; Saulnier & Thibault, 1999). The FA esters can be oxidatively coupled to form dimers as well as trimers, producing AX crosslinks within the cell wall (Mitchell *et al*., 2007; Buanafina *et al*., 2008). FA can also form links between AX and lignin (Ralph *et al*., 2004; Bartley *et al*., 2013). This crosslinking through FA has important functions, such as controlling the ability of the cell wall to extend, protection against pathogen attack, and inhibition of cell wall degradation by microorganisms and ruminants as well as cellulase digestion (Bartley *et al*., 2013). Xylosyl residues in xylan that are not decorated with sugars are often acetylated in the C2 or C3 positions, and in Arabidopsis the patterns of xylan substitution have been shown to contribute to xylan conformation and its interactions with cellulose (Busse‐Wicher *et al*., 2016), and altering xylan acetylation leads to changes in stem digestibility (Pawar *et al*., 2016).

Genetic engineering to improve biomass digestibility requires knowledge of the genetic factors that determine recalcitrance. Methods used for reducing biomass recalcitrance in grasses have involved reverse genetic approaches such as overexpression or RNA interference (RNAi) techniques of possible genes or transcription factors that play a role in cell wall biosynthesis (Bhatia *et al*., 2017). Quantitative trait analysis in recombinant inbred populations provides a powerful tool for identifying such genetic determinants based on linkage disequilibrium. Such studies can identify so‐called quantitative trait loci (QTLs), which are regions of the genome harbouring polymorphisms that cause quantitative variation in the trait of interest. QTL studies have been used to investigate animal feed digestibility to determine the major factors affecting this trait in maize (Cardinal *et al*., 2003; Courtial *et al*., 2014; Barrière *et al*., 2015). In the work presented here, we used a population of recombinant inbred lines (RILs) of the model grass *Brachypodium dystachyon* (Brachypodium) to look for QTLs for digestibility by measuring the saccharification potential of stems (susceptibility to digestion with commercial cellulases). Our data revealed a single QTL associated with saccharification and found that the most plausible candidate gene responsible for the variation in stem digestibility in this population encodes a putative GT43 protein. The putative role of this gene was supported by RNAi gene silencing to produce plants with reduced expression levels, which exhibited increased saccharification accompanied by modest changes in xylan content in their cell walls.

## Materials and Methods

### Plant material and growth conditions

A *Brachypodium distachyon* (Brachypodium) RIL population Bd3‐1 × Bd21 (Cui *et al*., 2012) was sown for QTL analysis in three randomized replicates (blocks 1–3) containing 12 plants per line within each replicate. The seeds were vernalized in the dark at 4°C for 3 wk before being transferred to the glasshouse where they grew under a long‐day, short‐night regime (16 : 8 h, light : dark) with temperatures ranging from 18 to 20°C. After 3 wk the plants were staked to help support the stems. Watering was stopped when the plants began to senesce and once they were completely dry the main stems were harvested. Brachypodium Bd21 and transgenic seeds were vernalized in the dark at 4°C for 1 wk before being transferred to the glasshouse and where they grew under the same conditions as the RIL population.

### Experimental design and statistical analysis

All analyses were conducted with the statistical software R v.3.2.3 (R Development Core Team, 2008). The experimental design was completely randomized replicated in three blocks. Data were adjusted to a lineal model with the function lm and factors effects were checked by an analysis of variance with the function ANOVA. The normality and heteroscedasticity assumptions were tested by plotting the residuals vs the predicted values and Q‐Q plots. The differences between lines were assessed using *post hoc* multiple‐comparison test (function glht, package multcomp) (Hothorn *et al*., 2008).

### Saccharification analysis

Saccharification analysis was performed on Brachypodium stem material which was prepared by removing the top and bottom internodes as well as all nodes. The main stem was selected and cut into 2 cm fragments and placed into 2 ml tubes together with two ball bearings and milled within the tube. The samples were formatted in 96‐well plates to contain four technical replicates of 4 mg each. The formatted 96‐well plates underwent saccharification analysis using a liquid handling platform which pretreated the samples with 0.5 N NaOH at 90°C for 30 min, followed by enzymatic hydrolysis at 50°C, pH 4.5 for 8 h. The enzyme cocktail contained commercially available Celluclast and Novozyme 188 (Novozymes A/S, Bagsvaerd, Denmark) at a ratio of 4 : 1. The reducing sugars released during hydrolysis were detected using a colorimetric assay involving 3‐methyl‐2‐benzothiazolinone hydrozone (MTBH) (Gomez *et al*., 2010, 2011).

### Quantitative trait detection

The saccharification data together with the genotype data (Cui *et al*., 2012) from the RIL Bd3‐1 × Bd21 population underwent QTL analysis following the method described in Broman & Sen (2009). A correction coefficient was applied to the saccharification data before QTL analysis to take into account any environmental effects caused by well position and sample weight. The command data=convert2riself(mydata) was used to include the algorithm for the investigation of a RIL population during the QTL analysis using the R/qtl program. Standard interval mapping was performed using a genome‐wide scan for the identification of loci. The significant threshold was determined using a 1000‐replicate permutation test and was displayed as a logarithm of the odds (LOD) 5% score. The QTL peak was selected as it exceeded this threshold. The QTL effect was obtained from an effect plot. The fit of the model was determined using the function fitqtl and 128 imputations with a 1 cM grid, which calculated the genetic variance of the QTL identified.

Broad‐sense heritability (*H* ^2^) was calculated from the value of the means squares of the RILs (Parker *et al*., 1998; Broman & Sen, 2009) as follows:

$$\begin{array}{c} H^{2}=V_{G}/V_{T}=V_{G}/\left(V_{G}+V_{E}\right) \end{array}$$

where *V*_G_ is the genotype variance, *V*_E_ is the environmental variance and *V*_T_ is the total variance of the trait of interest.

### Identification of candidate genes

GBrowser (http://mips.helmholtz-muenchen.de/gbrowse/plant/cgi-bin/gbrowse/brachy/) was used to investigate, *in silico*, the genomic region between the two markers flanking the QTL peak for possible candidate genes. The region on either side of marker BD1676.1 (physical position: 25 970 456 bp, according to the reference genome of Bd 21) on chromosome 5 was explored, therefore from marker BD4088.6 (physical position: 25889793 bp) to marker BD3488.1 (physical position: 2678751 bp).

### Phylogenetic tree

Protein sequences from *Arabidopsis thaliana*,* Nicotiana attenuate*,* Nicotianna tabacum*,* Oryza sativa* and *B. distachyon* were collated for the IRX subfamilies 9 and 14 from the National Center for Biotechnology Information and Phytozyme12 databases. All the sequences were uploaded into Mega6.0 (Tamura *et al*., 2013) and aligned using ClustalW. The phylogenetic analysis was conducted using the neighbour joining method with 2000 bootstrap replicates (Hall, 2008).

### Polymorphism detection in the candidate gene sequence

One hundred milligrams of green stems were harvested from 4‐wk‐old plants of both parental lines BD21 and Bd3‐1. Samples were flash‐frozen in liquid nitrogen before RNA extraction using the Qiagen RNeasy Mini kit (Qiagen). The quality and quantity of RNA were checked on a 1% agarose gel as well as a NanoDrop spectrophotometer (ThermoScientific, Loughborough, UK). The samples were diluted to 2 μg in 10 μl. The RNA was incubated for 5 min at 65°C together with 1 μl of 10 mM dNTPs and 1 μl oligo dT. cDNA was generated using the SuperScript II reverse transcriptase kit (Thermofisher, Stafford, UK) once the samples were at room temperature.

The target gene, Bradi5g24290.1, was amplified from the cDNA using the primers designed according to the specification of the cloning kit. The following primer sequences were used: GT43_F: 5′‐CAC CAT GAA GCT CCC GCT‐3′; GT43_R: 5′‐CTA GTG ACC ATC TTC AGT ATT TAC TAC G‐3′. The PCR products were cloned using the StrataClone Blunt PCR cloning kit and sequenced. BioEdit v.7.2.5 software was used to determine the presence of any single nucleotide polymorphism (SNPs) by comparing the sequences of the cloned parents to each other as well as to the mRNA sequence of Bradi5g25290.1 (NCBI accession: XM_010242235).

Sorting Intolerant From Tolerant (SIFT) analysis was conducted to determine effects on protein function due to the amino acid substitutions detected (http://sift.jcvi.org/www/SIFT_seq_submit2.html).

### Artificial microRNA construction

Artificial microRNA sequences were designed using the Web MicroRNA Designer platform (http://wmd3.weigelworld.org) and were based on the JGI Brachypodium genome annotation (The International Brachypodium Initiative, 2010). Constructs were engineered from the pBract214 plasmid to replace the targeting regions of the native Brachypodium microRNA precursor (Supporting Information Fig. S1; Table S1). MicroRNA targets were PCR‐amplified according to Warthmann *et al*. (2008) and cloned into the pCR^®^8⁄GW⁄TOPO^®^ TA Cloning Kit.

### Plant transformation

Transformation was carried out according to Vogel (2008) where seeds were collected from 6‐ to 7‐wk‐old plants and the gluma was removed. Surface sterilization of the seeds was conducted with a 1.3% NaClO solution containing 0.01% Triton‐X100 for 4 min. The embryos were dissected and placed on callus initiation medium. The calli were propagated for 7 wk with two subsequent subcultures at 4 and 6 wk following dissection. The 7‐wk‐old calli were immersed in an *A. tumefaciens* suspension for 5 min and dried on filter paper. The agrobacterium strain AGL1 was used together with the pBract 204 vectors which contain the hpt gene conferring hygromycin resistance under a 35s promoter at the left border (LB) and a gus gene encoding β‐glucuronidase under the control of the maize ubiquitin promoter at the right border (RB). The calli were then cocultivated on dry filter paper for 3 d in the dark at 22°C. Following cocultivation, the calli were moved to selective plates containing 40 mg l^−1^ hygromycin and 200 mg l^−1^ timentin and were left for 4 wk in the dark at 28°C. Following selection, they were moved to LS media for regeneration at 28°C under constant light and then onto MS media for root establishment. Finally, the plantlets were transplanted to soil and grown as previously described.

### Quantitative real‐time PCR

RNA was isolated from Brachypodium stems using TRIzol reagent (Invitrogen). An amount of RNA (0.4 μg) was subjected to a reverse transcription step using the high‐capacity cDNA archive kit (RevoScript RT PreMix Kit). Expression of the gene targeted for silencing was quantified by comparative quantitative real‐time PCR (qRT‐PCR), where 4 μl of cDNA was added to 7 μl of dH_2_O, 12.5 μl of 2X SYBR Green Master Mix (Applied Biosystems, Foster City, CA, USA) and 0.75 μl of 10 mM of each primer (Table S2). Three duplicate reactions were used for each sample, and each set included template controls containing water. The qRT‐PCR amplifications were conducted using an ABI Prism 7000 Sequence Detection System (Applied Biosystems, Foster City, CA, USA) under the following conditions: 10 min initial denaturation at 94°C and 40 cycles (94°C for 15 s; 60°C for 60 s) with a single fluorescent reading (SYBR Green I chemistry) at the end of each cycle. The qRT‐PCR data were normalized against the housekeeping genes ubiquitin‐conjugating enzyme 18 (UBC18) and S‐adenosylmethionine decarboxylase (SamDC).

### Cell wall polysaccharide composition analysis

Alcohol insoluble residue (AIR) was prepared as described by Fry (1988) with modifications. A total of 100 mg of ground stem material was incubated in phenol for 30 min at room temperature while shaking, followed by centrifugation at 3000 ***g*** for 10 min at 4°C. The supernatant was removed and the pellet was washed with the following solutions: twice with chloroform: methanol (1 : 1, v/v), twice with 80% (v/v) methanol, and once with 100% methanol. The pellets were left to dry overnight at room temperature. The samples were destarched by amylase treatment and 20 mg were suspended in 2 ml of 10 mM potassium phosphate buffer (pH 6.5), 1 mM CaCl_2_ and 0.05% NaN_3_. This suspension was heated at 95°C and the starch was allowed to gelatinize for 30 s before 1 U ml^−1^ thermostable α‐amylase (Megazyme, Leinster, Ireland). The suspension was incubated at 85°C for 15 min then cooled to 25°C before 10 U ml^−1^ amyloglucosidase and 1 U ml^−1^ pullulanase (Megazyme) were added. This solution was incubated for 16 h at 25°C with continuous shaking at 500 rpm. The suspension was centrifuged for 10 min at 6000 ***g*** and the supernatant was removed. The pellet was washed with 2 ml 10 mM potassium phosphate (pH 6.5), 1 mM CaCl_2_, 0.05% NaN_3_, centrifuged at 6000 ***g*** and the supernatant was discarded.

### Cell wall fractionation and determination of xylan molecular weight

Sequential extraction of xylan was performed by agitating 20 mg AIR in 2 ml 0.05 M trans‐1,2‐cyclohexanediaminetetraacetic acid (CDTA) (pH 6.5) for 24 h at room temperature. The suspension was centrifuged (14 000 ***g***, 4°C for 10 min) and the pellet washed once with deionized water. The supernatants were combined as the CDTA‐soluble fraction. The samples were subsequently extracted under oxygen‐free conditions using 0.05 M Na_2_CO_3_ containing 0.01 M NaBH_4_ for 24 h at 4°C to form the Na_2_CO_3_‐soluble fraction, 1 M KOH containing 0.01 M NaBH_4_ for 24 h at 4°C to form the 1 M KOH‐soluble fraction and 4 M KOH containing 0.01 M NaBH_4_ for 24 h at 4°C to form the 4 M KOH‐soluble fraction. All fractions were filtered through a GF/C glass fibre filter (Whatman). The Na_2_CO_3_ and KOH fractions were also chilled on ice and adjusted to pH 5 with glacial acetic acid. All cell wall fractions were then dialysed extensively against deionized water for 24 h and then lyophilized. The fractionation was repeated three times on three sets of plants grown independently and the mean of these three independent replicas was calculated. Analysis of the molecular weight of the xylan was conducted using a size‐exclusion chromatography (SEC) method (Brown *et al*., 2009). The 1 and 4 KOH fractions were separated by SEC and analysed with multi‐angle light‐scattering detector and a refractive index detector system. Fractions of both wild‐type and silenced lines were treated with xylanase and analysed in the same way. The data were analysed using the Astra V software and the molecular weights were estimated using the Zimm fit method with degree 1. The sample refractive index increment (d*n*/d*c*) used was 0.145.

### Monosaccharide profiling

Noncellulosic monosaccharide analysis was performed using high‐performance anion exchange chromatography (HPAEC) (Carbopac PA‐10; Dionex, Camberley, UK). AIR samples of 3 mg were hydrolysed with 1 ml of 2 M trifluoroacetic acid (TFA) for 4 h at 100°C, cooled to room temperature and evaporated completely. The pellet was rinsed twice with 200 μl isopropanol and resuspended in 100 μl deionized water. Samples were filtered with 0.45 μm polytetrafluoroethylene filters and separated by HPAEC as described in Jones *et al*. (2003). The separated monosaccharides were quantified using an external calibration containing seven monosaccharide standards at 100 μM (arabinose, fucose, galactose, glucose, mannose, rhamnose, and xylose) that were subjected to acid hydrolysis in parallel with the samples.

### *p*‐Coumaric and FA measurements

Ferulic acid in the cell was quantified according to Fry (1988). One millilitre of 1 M NaOH was added to 10 mg AIR and incubated under argon at 25°C in the dark for 24 h. After the addition of 2 M TFA, phenolics were partitioned twice in 1 ml butan‐1‐ol. The residue after evaporation was dissolved in 200 μl 50% methanol and filtered using 1 ml Strata‐X polymeric solid phase extraction columns (Phenomenex, Macclesfield, UK). The extract was analysed using high‐performance liquid chromatography on an activated reverse‐phase C18 5 μm (4.6 × 250 mm) XBridge column (Waters Inc., Wilmslow, UK) in 100% methanol‐5% acetic acid, with a 20–70% methanol gradient over 25 min at a flow rate of 2 ml min^−1^. FA was detected and quantified with a SpectraSYSTEM^®^ UV6000LP photodiode array detector (Thermo Scientific) and the UV‐visible spectra were collected at 240–400 nm and analysed against an FA standard.

## Results

### Screening a Brachypodium RIL population for saccharification potential reveals a single significant QTL

**Figure 1:**
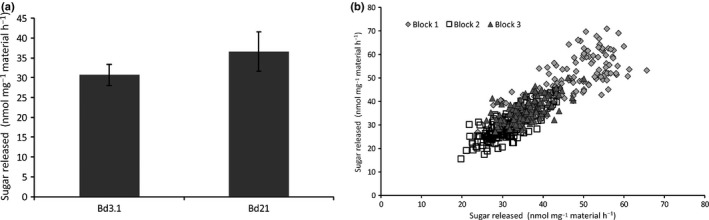
Saccharification analysis of Brachypodium stems. (a) Saccharification in straw from parental lines Bd3‐1 and Bd21. The ground material was digested with a commercial cellulase for 8 h at 50°C following a 0.5 N NaOH pretreatment of 30 min at 90°C. The results are the means ± SD of 48 replicates. (b) Distribution of the saccharification data from straw of three randomized replicate plots of the Brachypodium recombinant inbred line (RIL) population Bd21 × Bd3‐1. The ground material was digested with a commercial cellulase for 8 h at 50°C following a 0.5N NaOH pretreatment of 30 min at 90°C. The results are the means of the three plots containing 12 plants per line, which were analysed twice.

A Brachypodium F_6_ RIL population derived from a Bd21 × Bd3‐1 cross (Cui *et al*., 2012) was kindly supplied by David Garvin (University of Minnesota) and grown to maturity in a glasshouse in three independent blocks to provide straw for digestibility assays. The parental lines are significantly different from one another in terms of germination frequency and height, excluding the inflorescence, but showed similar total biomass yield (Fig. S2). Internodes from the stems of the plants were milled and subject to digestion with a commercial cellulase cocktail following a mild alkaline pretreatment as described previously (Gomez *et al*., 2010, 2011). These analyses showed that straw from the parental lines (Fig. 1a) had significant differences in digestibility. Bd21 straw showed higher saccharification potential (37 nmol sugar released mg^−1^ h^−1^) than Bd3‐1 (31 nmol mg^−1^ h^−1^). This difference in digestibility was shown to be statistically significant (two‐sample *t*‐test, *P* = 0.01).

The RIL population was analysed in three independent blocks and the results showed that the distribution of saccharification values within blocks 2 and 3 were similar, whereas block 1 showed a different distribution from these two (Fig. 1b). The plants grown in block 2 released on average the least amount of sugar (29.83 nmol mg^−1^ h^−1^) and block 1 released the most sugar (46.67 nmol mg^−1^ h^−1^), with block 3 releasing 35 nmol mg^−1^ h^−1^ on average.

**Figure 2:**
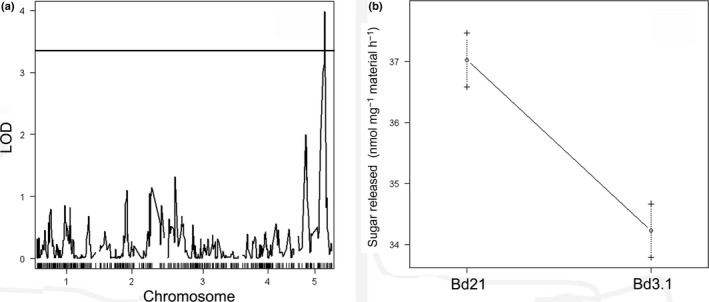
Quantitative trait locus (QTL) analysis of the recombinant inbred line (RIL) population. (a) Saccharification data from the RIL population showing a single peak, linked to straw digestibility, located on chromosome 5 (logarithm of the odds (LOD) 5% = 3.03). (b) Effect of the alleles on digestibility for the QTL linked to marker BD1676_1. Data are means ± SD.

QTL analysis was conducted using the saccharification data together with SNP data for the RIL population generated by Cui *et al*. (2012). QTL analysis conducted using R/qtl (Broman & Sen, 2009) identified a single QTL that exceeded the LOD 5% threshold of 3.3 on chromosome 5 linked to marker BD1676_1 (Fig. 2a).

It was calculated that this QTL accounts for 11.83% of the total variance observed for saccharification, allowing its classification as a major QTL (Prioul *et al*., 1999; Collard *et al*., 2005). The QTL resulted in an effect of −1.391 nmol sugar released mg^−1^ h^−1^ when comparing RIL bearing alleles from parental line AA with those bearing alleles from parental line BB (Fig. 2b); it also had a broad‐sense heritability (*H* ^2^) of 0.45.

The QTL linked to marker BD1676_1 was confirmed by selecting specific lines from the RIL population for further saccharification analysis. These lines were selected based on having either alleles from Bd21 or Bd3‐1 at marker BD1676_1. For this analysis, 24 lines were grown randomly in six replicates and analysed for digestibility using the same protocol as for the main population. The results revealed a significant difference (one‐way ANOVA, *P = *0.023) in saccharification between straw from lines carrying the alleles from AA (38.26 nmol sugar released mg^−1^ h^−1^) and the ones carrying the alleles from BB (36.13 nmol mg^−1^ h^−1^), giving independent support for the presence of the detected QTL for saccharification on this genomic region on chromosome 5.

### The QTL region contains a candidate family 43 glucosyltranferase gene

The genomic region around marker BD1676_1 (physical position 25 970 456 bp) was scrutinized between markers BD4088_6 (physical position 25 889 793 bp) and BD34881 (physical position 2647 871 bp) to identify candidate genes that could be responsible for variations in straw digestibility. A total of 104 genes are located in the examined region of the Brachypodium genome sequence v3.1 from the Phytozome12 database) (Table S3). Six candidate genes were selected for further *in silico* analysis based on known cell wall roles. The six genes included three WAK receptor‐like protein kinase subfamily B genes (*Bradi5g24180.2*,* Bradi5g24190.1* and *Bradi5g24310.1*) (Kohorn, 2001), a UDP‐galactosyltransferase (*Bradi5g24280.1*) (Edwards *et al*., 1999), a xylosyltransferase *GT43* family gene (*Bradi5g24290.1*) (Lee *et al*., 2012) and a UDP‐arabinopyranose mutase, *GT75*, gene (*Bradi5g24850.1*) (Zeng *et al*., 2010).

*In silico* gene expression data were analysed for each of these candidates across different libraries corresponding to different organs and developmental stages available from (2017) https://phytozome.jgi.doe.gov (Table 1). The expression levels for the *WAKs* and galactosyltransferase genes were low, particularly in stem libraries, while transcripts for the *GT43* and *GT75* genes are abundantly expressed in inflorescence stems, supporting a role in stem cell wall development. The profile of SNPs of *GT43* and *GT75* was examined to look for allelic differences between the genomic sequences of Bd21 and Bd3‐1 that might be related to the differences in saccharification observed. The genomic data showed the presence of a nonsynonymous SNP for GT43 and none for GT75 in the genome sequences of the parental lines (http://jbrowser.brachypodium.org). Based on these data we decided to focus our attention on the GT43 gene.

**Table 1:** The expression levels of candidate genes in the chromosome 5 region

	Candidate genes with cell wall functions											
	5g24180.2 (WAKb)		5g24190.1 (WAKb)		5g24310.1 (WAKb)		5g24280.1 (Galactosyltransferase)		5g24290.1 (GT43)		5g24850.1 (GT75)	
	FPKM	Locus DE	FPKM	Locus DE	FPKM	Locus DE	FPKM	Locus DE	FPKM	Locus DE	FPKM	Locus DE
Expression libraries at different developmental stages												
Flag leaf 47d 18lgt 6dk	0.181	ns	0.16	*	0.713	ns	0.19	ns	2.859	*	47.744	*
Flower 47d 18lgt 6dk	0.325	**	1.125	ns	0.655	ns	1.116	ns	31.991	**	151.81	**
Leaf mature 47d 18lgt 6dk	0.501	**	0.606	ns	0.983	ns	0.085	ns	3.451	ns	53.802	*
Leaf young 23d 18lgt 6dk	0.192	ns	0.701	ns	0.821	ns	0.181	ns	1.559	*	39.207	*
Shoot 24d 18lgt 6dk	0.166	ns	0.954	ns	0.836	ns	0.044	ns	4.058	ns	78.597	ns
Stem base 47d 18lgt 6dk	0.184	ns	1.874	ns	0.776	ns	0.052	ns	9.252	ns	165.28	**
Stem tip 47d 18lgt 6dk	0.158	ns	1.446	ns	0.785	ns	0.042	ns	14.591	**	136.98	**
Stem 47d 18lgt 6dk	0.096	*	1.074	ns	0.77	ns	0.085	ns	25.954	**	162.18	**

Data collected from selected *Brachypodium distachyon* v.3.1 expression libraries within the Phytozome database (https://phytozome.jgi.doe.gov). FPKM, fragments per kilobase of transcript per million mapped reads; Locus DE, for the gene, the expression level in this library is more than 1 SD above/below the average across all libraries; ns, not significant; *, significantly lower; **, significantly higher.

**Figure 3:**
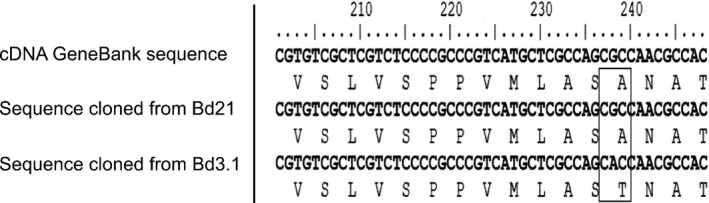
Sequence alignment of Bradi5g290.1 cloned from the parental lines, Bd21 and Bd3‐1 compared with wild‐type mRNA sequence (accession no. XM_010242235) from the National Center for Biotechnology Information database indicating a missense polymorphism.

The *GT43* gene, *Bradi5g24290.1*, was cloned from each parental line and sequenced to confirm the presence of SNPs that might account for the observed allelic variation in digestibility. Two SNPs were identified; the first was at a position of 111 bp from the start of the coding region and consisted of a change from a cytosine (C) to an adenine (A), which leads to no change in the encoded protein sequence. By contrast, the second SNP at position 238 altered a guanine (G) to an A, resulting in a missense variation leading to a change from an alanine at position 80 in the protein to a threonine in the sequence carried in the Bd3‐1 parental line (Fig. 3).

This change in amino acid occurs in a conserved region of the protein, and SIFT analysis indicates that a threonine is not tolerated at this position, as a score below 1.0 was returned in all databases analysed (Table S4) (Sim *et al*., 2012). Therefore, it is possible that this variation within the sequence of the gene could have an allelic effect on protein function that might impact on biomass digestibility and explain the presence of the detected QTL.

**Figure 4:**
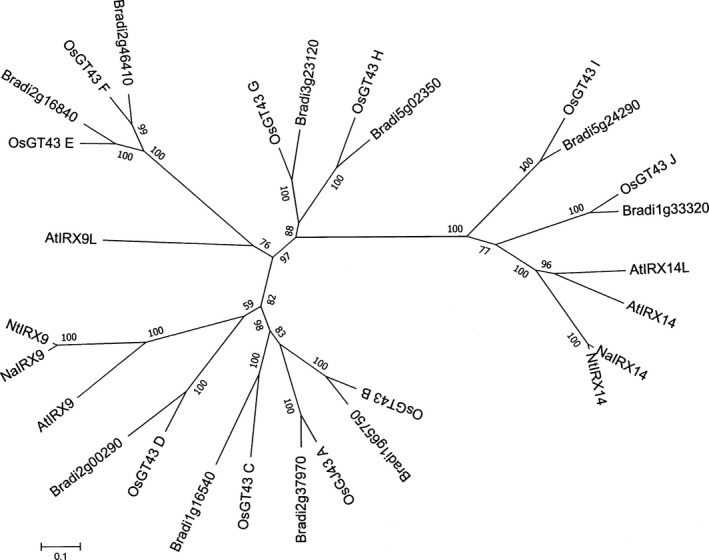
Phylogenetic analysis of the IRX9 and IRX14 proteins from Arabidopsis, tobacco, rice and Brachypodium. The sequence alignment was conducted using ClustalW and the phylogenetic analysis was done using the neighbour‐joining method in Mega 6.0. The bootstrap scores are from 2000 replicates and are shown on the nodes.

The Arabidopsis GT43 gene family involved in xylan synthesis consists of four members comprising two functionally nonredundant groups, IRX9 and its homologue IRX9L, as well as IRX14 and its homologue IRX14L (Wu *et al*., 2010). The Brachypodium genome contains 10 GT43 genes, the same number as reported in rice (Lee *et al*., 2014). The Brachypodium genes fall into clear orthologous groups along with those of rice, as determined by protein sequence phylogenetic analysis (Fig. 4). The *Bradi5 g24290.1* gene falls within the same clade as IRX14 genes from both Arabidopsis and rice, indicating that it is an IRX14 orthologue.

### RNAi gene suppression of the candidate *BdGT43A* leads to altered cell wall composition and increased saccharification

**Figure 5:**
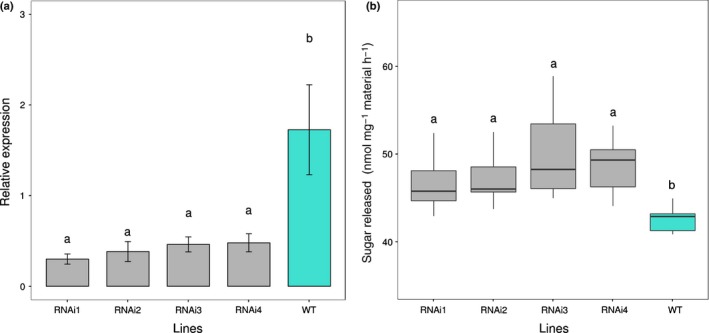
Analysis of the RNAinterference (RNAi) transgenic straw. (a) Transcript abundance indicated by the bar plot of the means for each level of independent variable of ANOVA showing ± SE. Tukey's honest significant difference (HSD) test indicates that those sharing the same letter are not significantly different. (b) Saccharification in silenced lines shown as boxplots to highlight the mean (line), 25^th^–75^th^ percentile (box) and 10^th^–90^th^ percentile (whiskers) of the glucose released for each genotype. Tukey's HSD test indicates that those sharing the same letter are not significantly different.

Transgenic RNAi gene‐silenced lines targeting *Bradi5g24290.1* were generated (Fig. S2; Table S3) and analysed to explore the effect of the knockdown of the candidate gene on cell wall composition, structure, and saccharification. The expression of the *Bd*GT43*A* gene was analysed in all transformants and four lines with expression levels of the gene reduced by *c*. 70% in stem tissue were characterized (Fig. 5a). Saccharification analysis using the same conditions as in the RIL population screening was conducted and the released glucose for the silenced lines was measured against nontransformed Bd21 as the wild‐type (Fig. 5b). An increase in saccharification compared with the wild‐type was observed in all four silenced lines, although only lines RNAi3 and RNAi4 showed a significant difference (Tukey's honest significant difference test). Interestingly, the transgenic plants showed no visible phenotype compared with the wild‐type.

**Figure 6:**
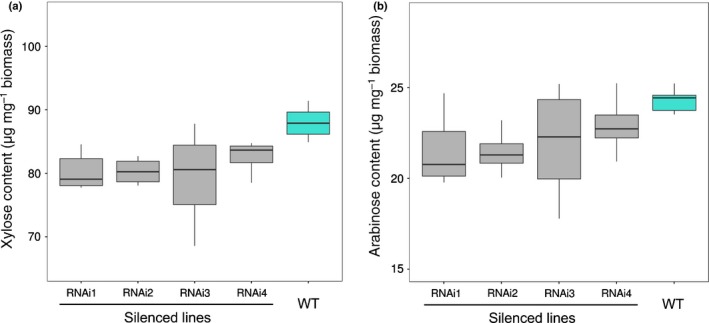
Amount of xylose (a) and arabinose (b) in the 1 M KOH cell wall fraction of silenced lines. The boxplots indicate the mean (line), 25^th^–75^th^ percentile (box) and 10^th^–90^th^ percentile (whiskers) for each genotype.

The cell walls of the transgenic plants and segregating wild‐types were further analysed to understand the underlying cause of the differences in saccharification. Stems from the transgenic and wild‐type plants were sequentially extracted with CDTA, Na_2_CO_3_, 1 M KOH and 4 M KOH to analyse the monosaccharide profile of matrix polysaccharide‐enriched fractions. The 1 M KOH cell wall fraction from mutant lines showed a statistically significant lower amount of xylose (Fig. 6a) and arabinose (Fig. 6b) compared with wild‐type plants.

**Figure 7:**
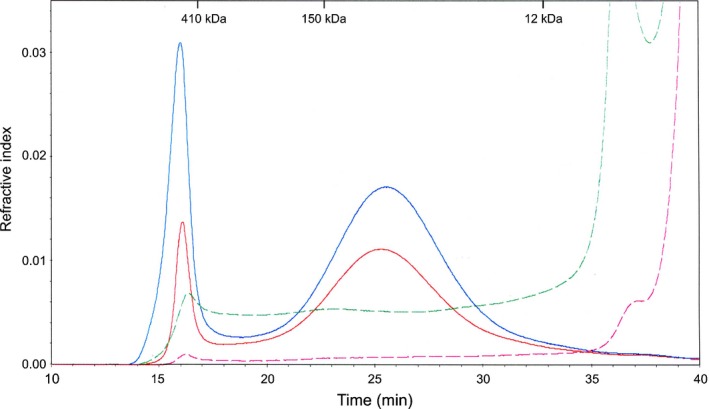
SECMALLS analysis to determine xylan chain length in the 1 M KOH fraction of cell walls from silenced lines. Blue line, wild‐type (WT) extracts; green dashed line, WT fraction treated with xylanase (control); red line, silenced lines; magenta dashed line, silenced control. Data shown are for the RNAi2, which is representative of the results obtained for all silenced lines. A 100 μl sample injection was used and data were analysed using AstraV software together with the Zimm Fit method to estimate the molecular weight. The sample refractive index increment was 0.145.

It was previously reported that *irx14* mutants in Arabidopsis showed a decrease in stem xylose content and that this was accompanied by shorter xylan backbones (Brown *et al*., 2007). The average chain length of xylans in the Brachypodium‐silenced lines was investigated using size exclusion chromatographic analysis, but no significant differences were observed, although the abundance of xylans in the 1 M KOH fraction from silenced lines is lower (Fig. 7).

**Figure 8:**
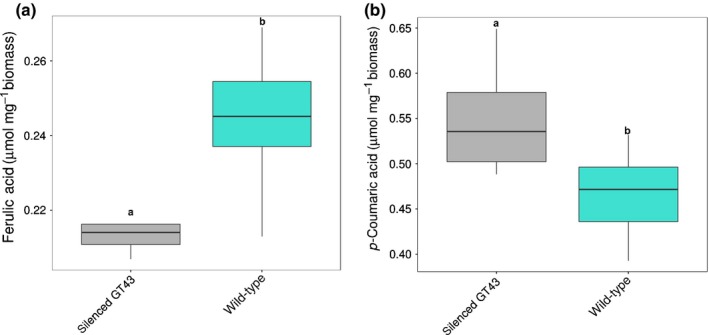
Amount of (a) ferulic acid and (b) *p‐*coumaric acid in wild‐type and the RNAi1 silenced line. The boxplots indicate the mean (line), 25^th^–75^th^ percentile (box) and 10^th^–90^th^ percentile (whiskers) for each genotype.

The amount of FA and *p*CA linked to arabinose residues in AX has been associated with the saccharification potential in grasses (Bartley *et al*., 2013). As the lines silenced for the *BdGT43A* gene show a small but significant reduction in arabinose, the FA and *p*CA content of the cell wall was analysed in stems of both transgenic and wild‐type plants. Transgenic lines showed a small but significant decrease in FA and an increase in *p*CA (Fig. 8a,b) when compared with the wild‐type.

## Discussion

Screening of the Brachpodium RIL population generated from a Bd3‐1 × Bd21 cross for straw saccharification revealed a single significant QTL for this characteristic on chromosome 5 (Fig. 2). Analysis of the QTL interval on chromosome 5 revealed several candidate genes with known functions in the cell wall, the most plausible of which encodes a *GT43* gene orthologue of *AtIRX14* (Fig. 4), which is involved in the biosynthesis of the xylan backbone in Arabidopsis (Wu *et al*., 2010). We tested if the *BdGT43A* gene is implicated in this process, by generating transgenic plants expressing RNAi gene suppression constructs, which showed an increase in straw saccharification compared with wild‐type plants (Fig. 5). Compositional analysis of cell wall matrix polysaccharides revealed decreased concentrations of xylose, arabinose and FA in the transgenic lines, suggesting that altered arabinoxylan composition was probably responsible for the increased digestibility of the straw.

The Brachypodium RIL population Bd3‐1 × Bd21 has been successfully used previously in the identification of the barley stripe mosaic virus resistant gene *bsr1* (Cui *et al*., 2012), QTLs for grass–pathogen interactions (Barbieri *et al*., 2012) and in understanding water‐use efficiency (Des Marais *et al*., 2016). The parental lines in this population showed a significant difference in saccharification potential (Fig. 1), allowing us to identify a single QTL for straw digestibility. A number of previous studies have identified QTLs for straw digestibility in rice (Dong *et al*., 2008; Liu *et al*., 2016), maize (Barrière *et al*., 2012), barley (Grando *et al*., 2005), sorghum (Wang *et al*., 2013) and *Miscanthus* (Van der Weijde *et al*., 2017). None of these previous studies succeeded in identifying and validating the causative genes and SNPs.

A QTL for saccharification was detected on chromosome 5 (Fig. 2). This QTL accounted for 11.83% of the total variance observed for saccharification within the population, which classifies it as a major QTL (Prioul *et al*., 1999; Collard *et al*., 2005). The heritability value for saccharification was 0.45, which is a slightly lower than that reported for saccharification as 0.53 in *Miscanthus* (Van der Weijde *et al*., 2017). Saccharification is a trait determined by a large number of environmental factors. Indeed, various lignocellulosic traits in maize including neutral detergent fibre (NDF), acid detergent lignin (ADL) and acid detergent fibre (ADF), have higher values (0.92, 0.74 and 0.92, respectively) (Karkowsky *et al*., 2005). In this study we have identified a single QTL related to saccharification potential. In *Miscanthus*, using the same method, seven QTLs were identified (Van der Weijde *et al*., 2017). There are a number of possible reasons for this low detection rate. First, the population size was relatively small and therefore only major QTLs could be detected (Parker *et al*., 1998). Second, although the parental lines have a significant difference in digestibility, the value of this difference is relatively small (Tan *et al*., 2001). In a recent study in rice we also detected a single QTL for saccharification, and observed that the parental lines, although not deviating enough from the median of the population, produced a progeny showing a much higher variation due to new combinations of interacting loci (Liu *et al*., 2016).

*GT43* genes have been characterized in plants as causing an *irregular xylem* (*irx*) phenotype initially described in Arabidopsis as secondary cell wall mutants, some of them associated with decreased xylan content (Brown *et al*., 2005). The *Bradi5 g24290.1* gene family members have been suggested as putative β‐1,4‐xylan‐synthases by Mitchell *et al*. (2007). *Bradi5g24290.1* RNAi gene‐silenced lines showed expression levels that were *c*. 75% below the wild‐type, and stem saccharification was higher than in the wild‐type. This increase in saccharification observed in the transgenic lines strongly supported the selection of *Bd*GT43A as the gene responsible for the QTL observed in chromosome 5, and was accompanied by a decrease in stem cell wall xylose content, evident in a 1 M KOH extracted fraction.

We could find little or no significant difference in the cell wall composition of our inbred lines carrying the alternate allelic forms. However, transgenic plants in which the expression of *BdGT43A* was suppressed showed a stronger saccharification phenotype than the RILs. In addition to decreased xylose content, the Bradi5g24290.1‐silenced lines showed a significant decrease in arabinose in the 1 M KOH cell wall fraction, most likely reflecting an overall decrease in AX.

In grasses, α‐(1‐3)‐linked arabinofuranosyl substitutions can be esterified with FA or *p*CA. FA can form crosslinks with other AX chains or with lignin (Sun *et al*., 1996, 2003). The rice *GT61* gene, *XAX1*, has been shown to have decreased FA as well as xylose when expression is disrupted, and this is accompanied by an increase in stem digestibility (Chiniquy *et al*., 2012). XAX1 appears to introduce xylosyl side chains associated with arabinosyl residues that carry FA esters, and the xax1 mutant exhibits decreased concentrations of FA in its AX; a similar phenotype is reported in the Brachypodium *sac1* mutant, which may be an orthologue of the rice *XAX1* gene (Marriott *et al*., 2014). It is unclear what is involved in this change in FA content, but it has been proposed that this is produced because the FA is only added to the arabinose side chain once the xylosyl residues are in position (Chiniquy *et al*., 2012).

Overexpression of the rice acyltransferase *OsAT10*, involved in the addition of hydroxycinnamates to arabinoxylans, leads to a decrease in FA and an increase in *p*CA, exposing a possible common regulation of both phenolics (Bartley *et al*., 2013), and results in higher stem digestibility. A similar effect is observed in our *Bd*GT43*A*‐silenced lines, where a decrease in the AX content is associated with lower FA content. Our results reaffirm the importance of AX in determining lignocellulose digestibility in grasses. Interestingly, we compared the saccharification of stems from Arabidopsis *irx14* mutants with their wild‐type equivalents, but found no increased saccharification (Fig. S3), although these mutants have significantly lower xylan content than wild‐type Arabidopsis (Brown *et al*., 2005). Arabidopsis xylans lack the extensive arabinosylation seen in grass xylans, as well as the associated FA esters. This suggests that it is the lower concentrations of FA seen in the *Bradi5 g24290.1* gene suppression lines that are responsible for the increased saccharification of their stems.

## Author contributions

C.W. analysed the RIL population and performed biochemical analysis. F.J.O.G. analysed the silenced lines and performed biochemical analysis. Both authors contributed equally to the experimental work and drafting of the manuscript. M.R. supervised the analysis of the population. R.S. provided technical support with the saccharification and biochemical work. A.D., S.G.A. and F.P. designed, supervised and carried out the generation of the transgenic lines. L.D.G. and S.J.M‐M. designed the research and contributed to the preparation of the manuscript. C.W. and F.J.O.G. contributed equally to this work.

## Supporting information

Please note: Wiley Blackwell are not responsible for the content or functionality of any Supporting Information supplied by the authors. Any queries (other than missing material) should be directed to the *New Phytologist* Central Office.

**Fig. S1** Sequence and map of the silencing construct.
**Fig. S2** Comparison of Brachypodium parental lines Bd21 and Bd3‐1.
**Fig. S3** Saccharification analysis of Arabidopsis Col.0 and T‐DNA line GT43.
**Table S1** Primers used during construction of the RNAi lines
**Table S2** Primers used for qPCR in the RNAi lines
**Table S3** Genes identified on chromosome 5 around the QTL linked to marker BD1676_1
**Table S4** SIFT analysis of the changes produced by the SNP in *Bradi5g24290.1*
