# Supplementary material for: A glycosyl transferase family 43 protein involved in xylan biosynthesis is associated with straw digestibility in Brachypodium distachyon
Source: New Phytol. 2018 Mar 25;218(3):974–85. doi: 10.1111/nph.15089 (PMC5947151; doi:10.1111/nph.15089)
Supplement: Supplementary file 1 — Fig. S1 Sequence and map of the silencing construct. Fig. S2 Comparison of Brachypodium parental lines Bd21 and Bd3‐1. Fig. S3 Saccharification analysis of Arabidopsis Col.0 and T‐DNA line GT43. Table S1 Primers used during construction of the RNAi lines Table S2 Primers used for qPCR in the RNAi lines Table S3 Genes identified on chromosome 5 around the QTL linked to marker BD1676_1 Table S4 SIFT analysis of the changes produced by the SNP in Bradi5g24290.1 [file NPH-218-974-s001.pdf]

***New Phytologist* Supporting Information**

Article title: **Mapping Quantitative Trait Loci for straw digestibility in *Brachypodium distachyon* implicates a gene encoding a glycosyl transferase family 43 protein involved in xylan biosynthesis.**

Authors: Caragh Whitehead[1*], Francisco J. Ostos Garrido[2*], Matthieu Reymond[3], Rachael Simister[1], Assaf Distelfeld[4], Sergio G. Atienza[2], Fernando Piston[2], Leonardo D. Gomez[1] and Simon McQueen-Mason[1].

Article acceptance date: 5 February 2018

The following Supporting Information is available for this article:

Fig. S1 Sequence and map of the silencing construct.

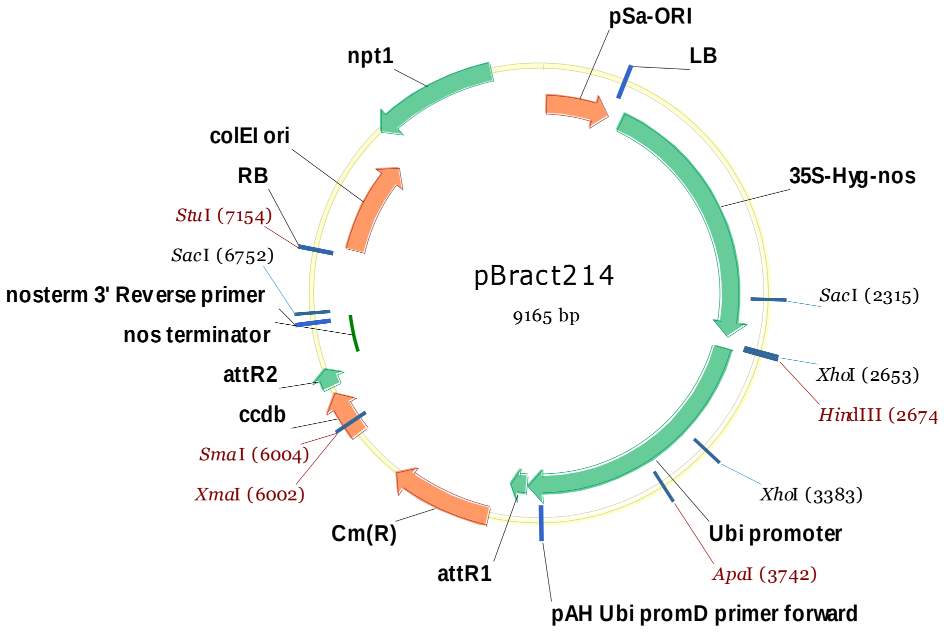

1 TTTTTATCCC CGGAAGCCTG TGGATAGAGG GTAGTTATCC ACGTGAAACC GCTAATGCCC

61 CGCAAAGCCT TGATTCACGG GGCTTTCCGG CCCGCTCCAA AAACTATCCA CGTGAAATCG

121 CTAATCAGGG TACGTGAAAT CGCTAATCGG AGTACGTGAA ATCGCTAATA AGGTCACGTG

181 AAATCGCTAA TCAAAAAGGC ACGTGAGAAC GCTAATAGCC CTTTCAGATC AACAGCTTGC

241 AAACACCCCT CGCTCCGGCA AGTAGTTACA GCAAGTAGTA TGTTCAATTA GCTTTTCAAT

301 TATGAATATA TATATCAATT ATTGGTCGCC CTTGGCTTGT GGACAATGCG CTACGCGCAC

361 CGGCTCCGCC CGTGGACAAC CGCAAGCGGT TGCCCACCGT CGAGCGCCAG CGCCTTTGCC

```
421   CACAACCCGG CGGCCGGCCG CAACAGATCG TTTTATAAAT TTTTTTTTTT GAAAAAGAAA
                                                                LB
                                                                ~
481   AAGCCCGAAA GGCGGCAACC TCTCGGGCTT CTGGATTTCC GATCCCCGGA ATTAGATCTT
               LB
      ~~~~~~~~~~~~~~~~~~~~~~~~~
541   GGCAGGATAT ATTGTGGTGT AACGTATCAC AAGTTTGTAC AAAAAAGCAG GCTCCGCGGC
601   CGCCCCCTTC ACCTAGACTC GACGCGTCCT AGAGATCCGT CAACATGGTG GAGCACGACA
661   CTCTCGTCTA CTCCAAGAAT ATCAAAGATA CAGTCTCAGA AGACCAAAGG GCTATTGAGA
721   CTTTTCAACA AAGGGTAATA TCGGGAAACC TCCTCGGATT CCATTGCCCA GCTATCTGTC
781   ACTTCATCAA AAGGACAGTA GAAAAGGAAG GTGGCACCTA CAAATGCCAT CATTGCGATA
841   AAGGAAAGGC TATCGTTCAA GATGCCTCTG CCGACAGTGG TCCCAAAGAT GGACCCCCAC
901   CCACGAGGAG CATCGTGGAA AAAGAAGACG TTCCAACCAC GTCTTCAAAG CAAGTGGATT
961   GATGTGATAT CTCCACTGAC GTAAGGGATG ACGCACAATC CCACTATCCT TCGCAAGACC
1021  CTTCCTCTAT ATAAGGAAGT TCATTTCATT TGGAGAGGAC GACCCCGATA TGAAAAAGCC
1081  TGAACTCACC GCGACGTCTG TCGAGAAGTT TCTGATCGAA AAGTTCGACA GCGTCTCCGA
1141  CCTGATGCAG CTCTCGGAGG GCGAAGAATC TCGTGCTTTC AGCTTCGATG TAGGAGGGCG
1201  TGGATATGTC CTGCGGGTAA ATAGCTGCGC CGATGGTTTC TACAAAGATC GTTATGTTTA
1261  TCGGCACTTT GCATCGGCCG CGCTCCCGAT TCCGGAAGTG CTTGACATTG GGGAATTCAG
1321  CGAGAGCCTG ACCTATTGCA TCTCCCGCCG TGCACAGGGT GTCACGTTGC AAGACCTGCC
1381  TGAAACCGAA CTGCCCGCTG TTCTGCAGGT AAATTTCTAG TTTTTCTCCT TCATTTTCTT
1441  GGTTAGGACC CTTTTCTCTT TTTATTTTTT TGAGCTTTGA TCTTTCTTTA AACTGATCTA
1501  TTTTTTAATT GATTGGTTAT GGTGTAAATA TTACATAGCT TTAACTGATA ATCTGATTAC
1561  TTTATTTCGT GTGTCTATGA TGATGATGAT AACTGCAGCC GGTCGCGGAG GCCATGGATG
1621  CGATCGCTGC GGCCGATCTT AGCCAGACGA GCGGGTTCGG CCCATTCGGA CCGCAAGGAA
1681  TCGGTCAATA CACTACATGG CGTGATTTCA TATGCGCGAT TGCTGATCCC CATGTGTATC
1741  ACTGGCAAAC TGTGATGGAC GACACCGTCA GTGCGTCCGT CGCGCAGGCT CTCGATGAGC
1801  TGATGCTTTG GGCCGAGGAC TGCCCCGAAG TCCGGCACCT CGTGCACGCG GATTTCGGCT
1861  CCAACAATGT CCTGACGGAC AATGGCCGCA TAACAGCGGT CATTGACTGG AGCGAGGCGA
1921  TGTTCGGGGA TTCCCAATAC GAGGTCGCCA ACATCTTCTT CTGGAGGCCG TGGTTGGCTT
1981  GTATGGAGCA GCAGACGCGC TACTTCGAGC GGAGGCATCC GGAGCTTGCA GGATCGCCGC
2041  GGCTCCGGGC GTATATGCTC CGCATTGGTC TTGACCAACT CTATCAGAGC TTGGTTGACG
```

```
2101  GCAATTTCGA TGATGCAGCT TGGGCGCAGG GTCGATGCGA CGCAATCGTC CGATCCGGAG

2161  CCGGGACTGT CGGGCGTACA CAAATCGCCC GCAGAAGCGC GGCCGTCTGG ACCGATGGCT

2221  GTGTAGAAGT ACTCGCCGAT AGTGGAAACC GACGCCCCAG CACTCGTCCG AGGGCAAAGG

                                      SacI

                                     ~~~~~~~

2281  AATAGAGTAG ATGCCGACCG GGATCCGGAG AGCTCGAATT TCCCCGATCG TTCAAACATT

2341  TGGCAATAAA GTTTCTTAAG ATTGAATCCT GTTGCCGGTC TTGCGATGAT TATCATATAA

2401  TTTCTGTTGA ATTACGTTAA GCATGTAATA ATTAACATGT AATGCATGAC GTTATTTATG

2461  AGATGGGTTT TTATGATTAG AGTCCCGCAA TTATACATTT AATACGCGAT AGAAAACAAA

2521  ATATAGCGCG CAAACTAGGA TAAATTATCG CGCGCGGTGT CATCTATGTT ACTAGATCGG

2581  GAATTCATCG ATGATATCAG ATCAAGGGTG GGCGCGCCGA ACCAGCTTTC TTGTACAAAG

                   XhoI                  HindIII

                  ~~~~~~                 ~~~~~~

2641  TGGTGATCCC CCTCGAGGTC GACGGTATCG ATAAGCTTGA TATCGAATTC CGTGCAGCGT

2701  GACCCGGTCG TGCCCCTCTC TAGAGATAAT GAGCATTGCA TGTCTAAGTT ATAAAAAATT

2761  ACCACATATT TTTTTTGTCA CACTTGTTTG AAGTGCAGTT TATCTATCTT TATACATATA

2821  TTTAAACTTT ACTCTACGAA TAATATAATC TATAGTACTA CAATAATATC AGTGTTTTAG

2881  AGAATCATAT AAATGAACAG TTAGACATGG TCTAAAGGAC AATTGAGTAT TTTGACAACA

2941  GGACTCTACA GTTTTATCTT TTTAGTGTGC ATGTGTTCTC CTTTTTTTTT GCAAATAGCT

3001  TCACCTATAT AATACTTCAT CCATTTTATT AGTACATCCA TTTAGGGTTT AGGGTTAATG

3061  GTTTTTATAG ACTAATTTTT TTAGTACATC TATTTTATTC TATTTTAGCC TCTAAATTAA

3121  GAAAACTAAA ACTCTATTTT AGTTTTTTTA TTTAATAATT TAGATATAAA ATAGAATAAA

3181  ATAAAGTGAC TAAAAATTAA ACAAATACCC TTTAAGAAAT TAAAAAAACT AAGGAAACAT

3241  TTTTCTTGTT TCGAGTAGAT AATGCCAGCC TGTTAAACGC CGTCGACGAG TCTAACGGAC

3301  ACCAACCAGC GAACCAGCAG CGTCGCGTCG GGCCAAGCGA AGCAGACGGC ACGGCATCTC

                              XhoI

                             ~~~~~~

3361  TGTCGCTGCC TCTGGACCCC TCTCGAGAGT TCCGCTCCAC CGTTGGACTT GCTCCGCTGT

3421  CGGCATCCAG AAATTGCGTG TCGGACGGCA GACGTGAGCC GGCACGGCAG GCGGCCTCCT

3481  CCTCCTCTCA CGGCACCGGC AGCTACGGGG GATTCCTTTC CCACCGCTCC TTCGCTTTCC

3541  CTTCCTCGCC CGCCGTAATA AATAGACACC CCCTCCACAC CCTCTTTCCC CAACCTCGTG

3601  TTGTTCGGAG CGCACACACA CACAACCAGA TCTCCCCCAA ATCCACCCGT CGGCACCTCC
```

```
3661  GCTTCAAGGT ACGCCGCTCG TCCTCCCCCC CCCCCCCTCT CTACCTTCTC TAGATCGGCG

                      ApaI

                     ~~~~~~~

3721  TTCCGGTCCA TGGTTAGGGC CCGGTAGTTC TACTTCTGTT CATGTTTGTG TTAGATCCGT

3781  GTTTGTGTTA GATCCGTGCT GCTAGCGTTC GTACACGGAT GCGACCTGTA CGTCAGACAC

3841  GTTCTGATTG CTAACTTGCC AGTGTTTCTC TTTGGGGAAT CCTGGGATGG CTCTAGCCGT

3901  TCCGCAGACG GGATCGATTT CATGATTTTT TTTTGTTTCG TTGCATAGGG TTTGGTTTGC

3961  CCTTTTCCTT TATTTCAATA TATGCCGTGC ACTTGTTTGT CGGGTCATCT TTTCATGCTT

4021  TTTTTTGTCT TGGTTGTGAT GATGTGGTCT GGTTGGGCGG TCGTTCTAGA TCGGAGTAGA

4081  ATTAATTCTG TTTCAAACTA CCTGGTGGAT TTATTAATTT TGGATCTGTA TGTGTGTGCC

4141  ATACATATTC ATAGTTACGA ATTGAAGATG ATGGATGGAA ATATCGATCT AGGATAGGTA

4201  TACATGTTGA TGCGGGTTTT ACTGATGCAT ATACAGAGAT GCTTTTTGTT CGCTTGGTTG

4261  TGATGATGTG GTGTGGTTGG GCGGTCGTTC ATTCGTTCTA GATCGGAGTA GAATACTGTT

4321  TCAAACTACC TGGTGTATTT ATTAATTTTG GAACTGTATG TGTGTGTCAT ACATCTTCAT

4381  AGTTACGAGT TTAAGATGGA TGGAAATATC GATCTAGGAT AGGTATACAT GTTGATGTGG

4441  GTTTTACTGA TGCATATACA TGATGGCATA TGCAGCATCT ATTCATATGC TCTAACCTTG

4501  AGTACCTATC TATTATAATA AACAAGTATG TTTTATAATT ATTTTGATCT TGATATACTT

              pAH Ubi_promD primer forward

              ~~~~~~~~~~~~~~~~~~~~~~~~~

4561  GGATGATGGC ATATGCAGCA GCTATATGTG GATTTTTTTA GCCCTGCCTT CATACGCTAT

4621  TTATTTGCTT GGTACTGTTT CTTTTGTCGA TGCTCACCCT GTTGTTTGGT GTTACTTCGC

4681  CCATCACAAG TTTGTACAAA AAAGCTGAAC GAGAAACGTA AAATGATATA AATATCAATA

4741  TATTAAATTA GATTTTGCAT AAAAAACAGA CTACATAATA CTGTAAAACA CAACATATCC

4801  AGTCACTATG GCGGCCGCAT TAGGCACCCC AGGCTTTACA CTTTATGCTT CCGGCTCGTA

4861  TAATGTGTGG ATTTTGAGTT AGGATCCGTC GAGATTTTCA GGAGCTAAGG AAGCTAAAAT

4921  GGAGAAAAAA ATCACTGGAT ATACCACCGT TGATATATCC CAATGGCATC GTAAAGAACA

4981  TTTTGAGGCA TTTCAGTCAG TTGCTCAATG TACCTATAAC CAGACCGTTC AGCTGGATAT

5041  TACGGCCTTT TTAAAGACCG TAAAGAAAAA TAAGCACAAG TTTTATCCGG CCTTTATTCA

5101  CATTCTTGCC CGCCTGATGA ATGCTCATCC GGAATTCCGT ATGGCAATGA AAGACGGTGA

5161  GCTGGTGATA TGGGATAGTG TTCACCCTTG TTACACCGTT TTCCATGAGC AAACTGAAAC

5221  GTTTTCATCG CTCTGGAGTG AATACCACGA CGATTTCCGG CAGTTTCTAC ACATATATTC

5281  GCAAGATGTG GCGTGTTACG GTGAAAACCT GGCCTATTTC CCTAAAGGGT TTATTGAGAA
```

5341 TATGTTTTTC GTCTCAGCCA ATCCCTGGGT GAGTTTCACC AGTTTTGATT TAAACGTGGC

5401 CAATATGGAC AACTTCTTCG CCCCCGTTTT CACCATGGGC AAATATTATA CGCAAGGCGA

5461 CAAGGTGCTG ATGCCGCTGG CGATTCAGGT TCATCATGCC GTTTGTGATG GCTTCCATGT

5521 CGGCAGAATG CTTAATGAAT TACAACAGTA CTGCGATGAG TGGCAGGGCG GGGCGTAAAC

5581 GCGTGGATCC GGCTTACTAA AAGCCAGATA ACAGTATGCG TATTTGCGCG CTGATTTTTG

5641 CGGTATAAGA ATATATACTG ATATGTATAC CCGAAGTATG TCAAAAAGAG GTATGCTATG

5701 AAGCAGCGTA TTACAGTGAC AGTTGACAGC GACAGCTATC AGTTGCTCAA GGCATATATG

5761 ATGTCAATAT CTCCGGTCTG GTAAGCACAA CCATGCAGAA TGAAGCCCGT CGTCTGCGTG

5821 CCGAACGCTG GAAAGCGGAA AATCAGGAAG GGATGGCTGA GGTCGCCCGG TTTATTGAAA

5881 TGAACGGCTC TTTTGCTGAC GAGAACAGGG GCTGGTGAAA TGCAGTTTAA GGTTTACACC

5941 TATAAAAGAG AGAGCCGTTA TCGTCTGTTT GTGGATGTAC AGAGTGATAT TATTGACACG

XmaI

~~~~~~

SmaI

~~~~~~

6001 CCCGGGCGAC GGATGGTGAT CCCCCTGGCC AGTGCACGTC TGCTGTCAGA TAAAGTCTCC

6061 CGTGAACTTT ACCCGGTGGT GCATATCGGG GATGAAAGCT GGCGCATGAT GACCACCGAT

6121 ATGGCCAGTG TGCCGGTCTC CGTTATCGGG GAAGAAGTGG CTGATCTCAG CCACCGCGAA

6181 AATGACATCA AAAACGCCAT TAACCTGATG TTCTGGGGAA TATAAATGTC AGGCTCCCTT

6241 ATACACAGCC AGTCTGCAGG TCGACCATAG TGACTGGATA TGTTGTGTTT TACAGTATTA

6301 TGTAGTCTGT TTTTTATGCA AAATCTAATT TAATATATTG ATATTTATAT CATTTTACGT

6361 TTCTCGTTCA GCTTTCTTGT ACAAAGTGGT GATGGGGGAT CCACTAGTTC TAGAATTCGA

6421 TTGAGTCAAG CAGGATCGTT CAAACATTTG GCAATAAAGT TTCTTAAGAT TGAATCCTGT

6481 TGCCGGTCTT GCGATGATTA TCATATAATT TCTGTTGAAT TACGTTAAGC ATGTAATAAT

6541 TAACATGTAA TGCATGACGT TATTTATGAG ATGGGTTTTT ATGATTAGAG TCCCGCAATT

6601 ATACATTTAA TACGCGATAG AAAACAAAAT ATAGCGCGCA AACTAGGATA AATTATCGCG

nosterm_3' Reverse primer

~~~~~~~~~~~~~~~~~~~~~~~~

6661 CGCGGTGTCA TCTATGTTAC TAGATCGACC GGCATGCAAG CTGATATCAA TCACTAGTGA

SacI

~~~~~~~

6721 ATTCTAGAGC GGCCGCCACC GCGGTGGAGC TCCAGCTTTT GTTCCCTTTA GTGAGGGTTA

```
6781  ATTGCGCGCT TGGCGTAATC ATGGTCATAG CTGTTTCCTG TGTGAAATTG TTATCCGCTC
6841  ACAATTCCAC ACAACATACG AGCCGGAAGC ATAAAGTGTA AAGCCTGGGG TGCCTAATGA
6901  GTGAGCTAAC TCACATTAAT TGCGTTGCGC TCACTGCCCG CTTTCCAGTC GGGAAACCTG
6961  TCGTGCCAGC TGCATTAATG AATCGGCCAA CGCGCGGGGA GAGGCGGTTT GCGTATTGGG
7021  CGCTCTTCCG CTTCCTCGCT CACTGACTCG CTGCGCTCGG TCGTTCGGCT GCGGCGAGCG
7081  GTATCAGCTC ACTCAAAGGC GGTAATACGG TTATCCACAG AATCAGGGGA TAACGCAGGA
                                       RB
                            ~~~~~~~~~~~~~~~~~~~~~~~~~
                 StuI
                ~~~~~~
7141  AAGAACATGA AGGCCTTGAC AGGATATATT GGCGGGTAAA CTAAGTCGCT GTATGTGTTT
7201  GTTTGAGATC TCATGTGAGC AAAAGGCCAG CAAAAGGCCA GGAACCGTAA AAAGGCCGCG
7261  TTGCTGGCGT TTTTCCATAG GCTCCGCCCC CCTGACGAGC ATCACAAAAA TCGACGCTCA
7321  AGTCAGAGGT GGCGAAACCC GACAGGACTA TAAAGATACC AGGCGTTTCC CCCTGGAAGC
7381  TCCCTCGTGC GCTCTCCTGT TCCGACCCTG CCGCTTACCG GATACCTGTC CGCCTTTCTC
7441  CCTTCGGGAA GCGTGGCGCT TTCTCATAGC TCACGCTGTA GGTATCTCAG TTCGGTGTAG
7501  GTCGTTCGCT CCAAGCTGGG CTGTGTGCAC GAACCCCCCG TTCAGCCCGA CCGCTGCGCC
7561  TTATCCGGTA ACTATCGTCT TGAGTCCAAC CCGGTAAGAC ACGACTTATC GCCACTGGCA
7621  GCAGCCACTG GTAACAGGAT TAGCAGAGCG AGGTATGTAG GCGGTGCTAC AGAGTTCTTG
7681  AAGTGGTGGC CTAACTACGG CTACACTAGA AGAACAGTAT TTGGTATCTG CGCTCTGCTG
7741  AAGCCAGTTA CCTTCGGAAG AAGAGTTGGT AGCTCTTGAT CCGGCAAACA AACCACCGCT
7801  GGTAGCGGTG GTTTTTTTGT TTGCAAGCAG CAGATTACGC GCAGAAAAAA AGGATCTCAA
7861  GAAGATCCTT TGATCTTTTC TACGGGGTCT GACGCTCAGT GGAACGAAAA CTCACGTTAA
7921  GGGATTTTGG TCATGAGATT ATCAAAAAGG ATCTTCACCT AGATCCTTTT AAATTAAAAA
7981  TGAAGTTTTA AATCAATCTA AAGTATATAT GTGTAACATT GGTCTAGTGA TTAGAAAAAC
8041  TCATCGAGCA TCAAATGAAA CTGCAATTTA TTCATATCAG GATTATCAAT ACCATATTTT
8101  TGAAAAAGCC GTTTCTGTAA TGAAGGAGAA AACTCACCGA GGCAGTTCCA TAGGATGGCA
8161  AGATCCTGGT ATCGGTCTGC GATTCCGACT CGTCCAACAT CAATACAACC TATTAATTTC
8221  CCCTCGTCAA AAATAAGGTT ATCAAGTGAG AAATCACCAT GAGTGACGAC TGAATCCGGT
8281  GAGAATGGCA AAAGTTTATG CATTTCTTTC CAGACTTGTT CAACAGGCCA GCCATTACGC
8341  TCGTCATCAA AATCACTCGC ATCAACCAAA CCGTTATTCA TTCGTGATTG CGCCTGAGCG
8401  AGACGAAATA CGCGATCGCT GTTAAAAGGA CAATTACAAA CAGGAATCGA ATGCAACCGG
```

8461 CGCAGGAACA CTGCCAGCGC ATCAACAATA TTTTCACCTG AATCAGGATA TTCTTCTAAT

8521 ACCTGGAATG CTGTTTTCCC TGGGATCGCA GTGGTGAGTA ACCATGCATC ATCAGGAGTA

8581 CGGATAAAAT GCTTGATGGT CGGAAGAGGC ATAAATTCCG TCAGCCAGTT TAGTCTGACC

8641 ATCTCATCTG TAACAACATT GGCAACGCTA CCTTTGCCAT GTTTCAGAAA CAACTCTGGC

8701 GCATCGGGCT TCCCATACAA TCGGTAGATT GTCGCACCTG ATTGCCCGAC ATTATCGCGA

8761 GCCCATTTAT ACCCATATAA ATCAGCATCC ATGTTGGAAT TTAATCGCGG CCTTGAGCAA

8821 GACGTTTCCC GTTGAATATG GCTCATAACA CCCCTTGTAT TACTGTTTAT GTAAGCAGAC

8881 AGTTTTATTG TTCATGATGA TATATTTTTA TCTTGTGCAA TGTAACATCA GAGATTTTGA

8941 GACACAACGT GGCTTTGTTG AATAAATCGA ACTTTTGCTG AGTTGAAGGA TCAGATCACG

9001 CATCTTCCCG ACAACGCAGA CCGTTCCGTG GCAAAGCAAA AGTTCAAAAT CACCAACTGG

9061 TCCACCTACA ACAAAGCTCT CATCAACCGT GGCTCCCTCA CTTTCTGGCT GGATGATGGG

9121 GCGATTCAGG CGATCCCCAT CCAACAGCCC GCCGTCGAGC GGGCT

Fig. S2 Comparison of the Brachypodium parental lines Bd21 and Bd3-1 in terms of (A) germination frequency (B) height, excluding inflorescence (C) total biomass and (D) stem only biomass. The results indicate the mean and +/- SD and Anova indicates that those sharing the same letter are not significantly different.

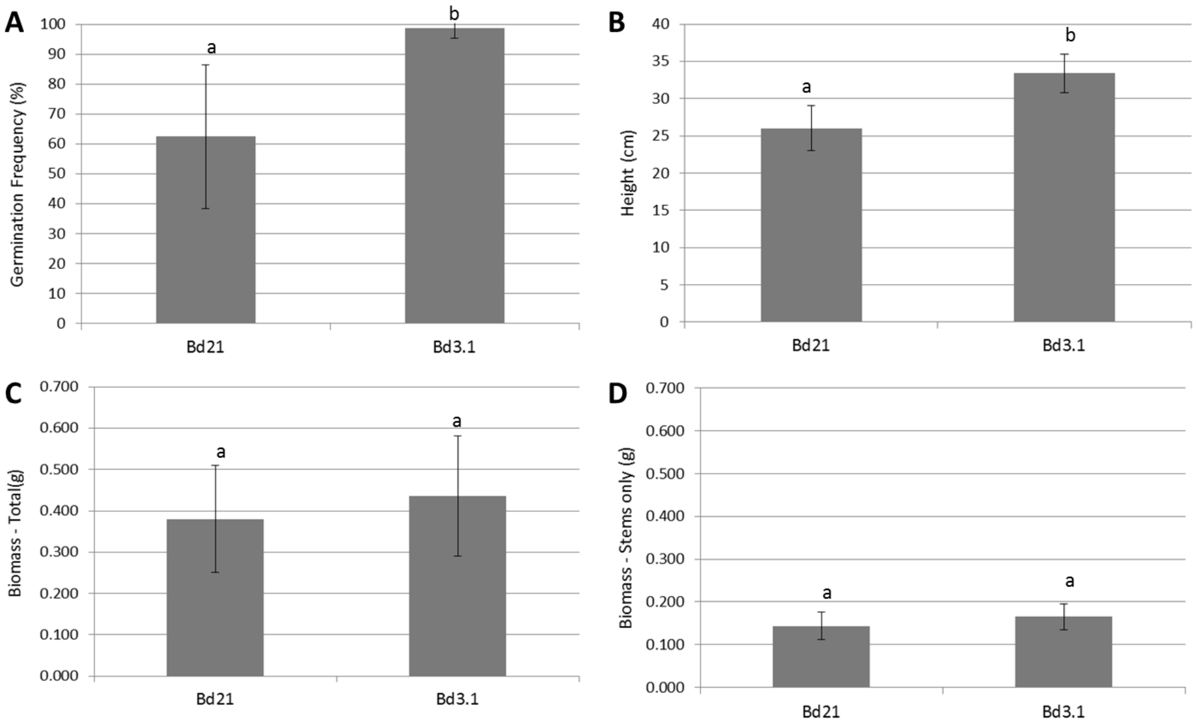

Fig. S3 Saccharification analysis of Arabidopsis Col.0 and T-DNA line GT43. The results indicate the mean and +/- SD.

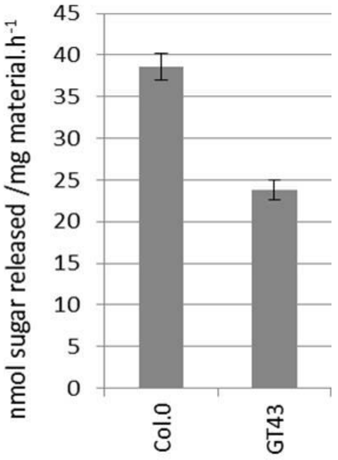

Table S1 Primers used during construction of the RNAi lines.

Oligo Name	Sequence 5' to 3'
miR-XS24290A-I	agTCTTCAGTATTTACTACGCTGcaggagattcagtttga
miR-XS24290A-II	tgCAGCGTAGTAAATACTGAAGActgctgctgctacagcc
miR*-XS24290A-III	ctCAGCGAAGTTAATACTGAAGAttcctgctgctaggctg
miR*-XS24290A-IV	aaTCTTCAGTATTAACTTCGCTGagagaggcaaaagtgaa
miR-XS24290B-I	agTTCAAATTTCCATACATGCGGcaggagattcagtttga
miR-XS24290B-II	tgCCGCATGTATGGAAATTTGAActgctgctgctacagcc
miR*-XS24290B-III	ctCCGCAAGTAAGGAAATTTGAAttcctgctgctaggctg
miR*-XS24290B-IV	aaTTCAAATTTCCTTACTTGCGGagagaggcaaaagtgaa
G-4368	CTGCAAGGCGATTAAGTTGGGTAAC
G-4369	GCGGATAACAATTTCACACAGGAAACAG

Table S2 Primers used during qPCR of the RNAi lines.

Oligo Name	Direction	Size (bp)	Sequence 5’ to 3’
SamDC	Forward	190	TGCTAATCTGCTCCAATGGC
	Reverse		GACGCAGCTGACCACCTAGA
UBC18	Forward	193	GGAGGCACCTCAGGTCATTT
	Reverse		ATAGCGGTCATTGTCTTGCG
Ubi10	Forward	237	TCCACACTCCACTTGGTGCT
	Reverse		GAGGGTGGACTCCTTTTGGA
BraXS90-RT-1	Forward	167	CACGCCCTCAGGGAGATACG
	Reverse		AACGACTCCTTGGTGCCTTC
BraXS90-RT-2	Forward	163	AGTCAAATTCCCGCATGAATGG
	Reverse		TCGGTGGAGGCTTCACAAAG

Table S3 Genes identified on chromosome 5 around the QTL linked to marker BD1676_1.

Gene Name		Function	Pfam	Panther
Bradi5g	24160.1	Motor activity, ATPase activity, Rab GTPase binding		GOLGIN-84
	24160.2	Motor activity, ATPase activity, Rab GTPase binding		GOLGIN-84
	24170.1	Secondary active sulfate transmembrane transporter activity, chloride channel activity, anion exchanger activity	Sulfate transporter family	Sulfate transporter
	24170.2	Secondary active sulfate transmembrane transporter activity, chloride channel activity, anion exchanger activity	Sulfate transporter family	Sulfate transporter
	24180.1	WAK receptor-like protein kinase, expressed, subfamily WAKb	Protein kinase domain	
	24190.1	WAK receptor-like protein kinase, expressed, subfamily WAKb	Protein kinase domain	
	24200.1	Putative Gene	Peroxidase	
	24207.1	Putative Gene	Alpha-N-acetylglucosaminidase (NAGLU)	Alpha-N-acetylglucosaminidase
	24220.1	Transcription corepressor activity, phosphoglycerate dehydrogenase activity		2-Hydroxyacid dehydrogenase
	24227.1	Putative Gene	Asparaginase	Protease T2 asparaginase
	24240.1	Putative Gene	Sec1 family	Vesicle protein sorting-associated
	24250.1	Single-standed DNA specific 3'-5' exodeoxyribonuclease activity	Exonuclease	Exonuclease-related
	24257.1	Putative Gene	DUF2930	
	24267.1	Putative Gene	Dedicator of cytokinesis	dedicator of cytokinesis (DOCK)
	24280.1	UDP-galactosyltransferase activity	UDP-glucoronosyl and UDP-glucosyl transferase	UDP-glucuronosyltransferase related

	24290.1	Putative xylosyltransferase, CAZy family GT43	Glycosyltransferase family 43	Beta-1,3-glucuronyltransferase-related
	24300.1	Serine-type carboxypeptidase activity	Serine carboxypeptidase	Serine carbosypeptidase II (carboxypeptidase D)
	24310.1	WAK receptor-like protein kinase, expressed, subfamily WAKb	Protein kinase domain	
	24320.1	Putative Gene		
	24330.1	Putative Gene		
	34340.1	Putative Gene		
	34350.1	Putative Gene		
	24360.1	Putative Gene	AP2 domain	Protein kinase
	24370.1	NADPH-hemoprotein reductase activity, iron ion binding, FAD binding, nitric-oxide synthase activity		NADPH-cytochrome P450 reductase
	24380.1	Putative Gene	AUX/IAA family	
	24387.1	Putative Gene	Sodium/calcium exchanger protein	
	24397.1	Putative Gene	Transmembrane proteins 14C	
	24410.1	Putative Gene	Tify domain	
	24410.2	Putative Gene	Tify domain	
	24420.1	Aminoacyl-tRNA hydrolase activity	Peptidyl-tRNA hydrolase PTH2	Peptidyl-tRNA hydrolase 2
	24430.1	Endonuclease activity	S1/P1 nuclease	
	24430.2	Endonuclease activity	S1/P1 nuclease	
	24440.1	Putative Gene		
	24450.1	Protein kinase family protein, putative, expressed, subfamily RLCK-VI	Protein tyrosine kinase	
	24460.1	Putative Gene	Calmodulin binding protein-like	
	24460.2	Putative Gene	Calmodulin binding protein-like	

	24460.3	Putative Gene	Calmodulin binding protein-like	
	24470.1	Metalloendopeptidase activity	Mov34/MPN/PAD-1 family	JUN activation domain binding protein
	24480.1	Protein kinase family protein, putative, subfamily, SD-2a	Protein kinase domain	
	24490.1	F-Box	F-Box	
	24490.2	F-Box	F-Box	
	24490.3	F-Box	F-Box	
	24500.1	Serine-type endopeptidase activity		Subtilinsin/Kexin-related serine protease
	24510.1	Putative Gene		
	24520.1	Serine-type endopeptidase activity	Peptidase inhibitor I9	Subtilinsin/Kexin-related serine protease
	24530.1	Protein serine/threonine phosphatase activity	Protein phosphatase 2C	Protein phosphatase 2c
	24536.1	Putative Gene		
	24542.1	Putative Gene	Cupin superfamily protein	MINA53 (MYC induced nuclear antigen
	24550.1	Glutamate-ammonia ligase activity, ATP binding	Glutamine synthetase	Glutamine synthetase
	24550.2	Glutamate-ammonia ligase activity, ATP binding	Glutamine synthetase	Glutamine synthetase
	24560.1	Putative Gene		
	24570.1	Protein kinase family protein, putative, subfamily, RLCK-OS1	Protein kinase domain	
	24580.1	Putative Gene	Nucleolar protein, Nop52	NNP-1 protein (novel nuclear protein 1, NOP52
	24590.1	ATP binding	ATPase family associated with various cellular activities (AAA)	
	24600.1	Putative Gene		
	24610.2	Glucose-6-phosphate 1-epimerase activity	Aldose 1-epimerase	Apospory-associated protein c-related
	24610.3	Glucose-6-phosphate 1-epimerase activity	Aldose 1-epimerase	Apospory-associated protein c-related

	24610.4	Glucose-6-phosphate 1-epimerase activity	Aldose 1-epimerase	Apospory-associated protein c-related
	24620.1	Putative Gene		
	24630.1	ATP binding	Lipase (class 3)	Alpha/beta hydrolase related
	24640.1	Protein farnesyltransferase activity	Polyprenyl synthetase	Farnesyl-prophosphate synthetase
	24640.2	Protein farnesyltransferase activity	Polyprenyl synthetase	Farnesyl-prophosphate synthetase
	24650.1	Putative Gene	Peroxidase	
	24660.1	Putative Gene	Universal stress protein family	
	24670.1	Putative Gene	SBP domain	
	24680.1	Putative Gene	Ribosomal protein L7Ae/L30e/S12e/Gadd45 family	60S Ribosomal protein 10A-related
	24690.1	Autoinhibited H+ P-type ATPase subfamily P3 cluster 2 from PMID:12805592. Similar to AtAHA1 plasma membrane H+ transporter		
	24700.1	Putative Gene	AP2 domain	
	24710.1	Putative Gene	AP2 domain	
	24720.1	Putative Gene	AP2 domain	
	24730.1	Protein kinase activity		
	24730.2	Protein kinase activity		
	24737.1	Putative Gene	Protein tyrosine kinase	
	24750.1	Protein kinase family protein, putative, expressed, subfamily RLCK-Os4	Protein tyrosine kinase	
	24760.1	Protein kinase family protein, putative, expressed, subfamily RLCK-Os4	Protein tyrosine kinase	
	24770.1	Putative Gene		
	24780.1	Serine-type endopeptidase activity		Subtilisin/Kexin-related Serine protease
	24790.1	BTB	BTB/POZ domain	

	24800.1	Neutral amino acid transmembrane transporter activity, L-amino acid transmembrane transporter activity	Transmembrane amino acid transporter protein	Amino acid transporter
	24810.1	Neutral amino acid transmembrane transporter activity, L-amino acid transmembrane transporter activity	Transmembrane amino acid transporter protein	Amino acid transporter
	24820.1	Endoribonuclease activity	eRF1 domain	PELOTA
	24830.1	Putative Gene		
	24836.1	Putative Gene	ABC1 family	ABC transporter-related
	24842.1	Putative Gene		
	24850.1	Similar to UDP-arabinopyranose mutase. Reversibly glycosylated polypeptide. CAZy family GT75	Reversibly glycosylated polypeptiede	
	24860.1	Putative Gene		
	24870.1	STE_MEKK_ste11_MAP3K.24 - STE kinases include homologs to sterile 7, sterile 11 and sterile 20 from yeast, expressed, subfamily	Protein kinase domain	
	24870.2	STE_MEKK_ste11_MAP3K.24 - STE kinases include homologs to sterile 7, sterile 11 and sterile 20 from yeast, expressed, subfamily	Protein kinase domain	
	24870.3	STE_MEKK_ste11_MAP3K.24 - STE kinases include homologs to sterile 7, sterile 11 and sterile 20 from yeast, expressed, subfamily	Protein kinase domain	
	24880.1	Putative Gene	Heavy-metal-associated domain	Copper transport protein ATOX1-related
	24890.1	Inositol pentakisphosphate 2-kinase activity, ATP binding	Inositol-pentakisphosphate 2-kinase	Inositol polyphosphate kinase 1
	24900.1	Putative Gene		
	24900.3	Putative Gene		
	24910.1	Putative Gene		
	24917.1	Putative Gene	Eukaryotic DNA topoisomerase I	DNA topoisomerase Type 1

	24930.1	Putative Gene		Molybdopterin biosynthesis protein
	24930.3	Putative Gene		Molybdopterin biosynthesis protein
	24930.4	Putative Gene		Molybdopterin biosynthesis protein
	24937.1	Putative Gene		
	24950.1	Transcription coactivator activity	MED7 protein	
	24950.3	Transcription coactivator activity	MED7 protein	
	24960.1	26S, subfamily 19S	Mov34/MPN/PAD-1 family	EIF3F-related
	24967.1	Putative Gene	BSD domain	
	24980.1	Putative Gene		40S Ribosomal protein S9

Table S4 SIFT analysis of the changes produced by the SNP in *Bradi5g24290.1*.

**Database**	**Predicted amino acid not tolerated**	**Number of sequences compared**	**Sequence represented**	**Predicted amino acid tolerated**
SwissPort	ywvtsrqpnmlki hgfedc	15	0.06	a
TrEMBL	whyfimqrndelc kvtp	54	0.16	gsa
UniRef90	whyfmiqrndelc kvtpg	61	0.1	sa
NCBI	Whyfimqrndelk cvtp	72	0.15	gsa
